# Historical Changes in Terminal Decline in Well-Being Among Japanese Older Adults

**DOI:** 10.1093/geroni/igaf122.663

**Published:** 2025-12-31

**Authors:** Takeshi Nakagawa, Gizem Hueluer, Denis Gerstorf, Jersey Liang, Erika Kobayashi

**Affiliations:** The University of Osaka, Osaka, Osaka, Japan; University of Bamberg, Bamberg, Germany; Humboldt University Berlin, Humboldt University Berlin, Berlin, Germany; University of Michigan, Ann Arbor, Michigan, United States; Tokyo Metropolitan Institute for Geriatrics and Gerontology, Itabashi City, Tokyo, Japan

## Abstract

Historical changes in psychosocial functioning at the end of life have been underexplored, and conflicting scenarios have been proposed regarding secular trends in the rate of decline: While the compression of morbidity hypothesis predicts a steeper rate of decline over historical time, the concept of “manufactured survival” suggests that the rate of decline has become shallower. This pre-registered study (https://osf.io/jd8xr) examined how individuals’ changes in well-being in the last years of life and their correlates differ over historical time. Data were derived from a longitudinal study of a nationally representative sample of Japanese older adults aged 60+ years. We compared two death-year cohorts based on individuals’ year of death (earlier: 1987-1999 versus later: 2000-2012) case-matched based on age at death and sex (n = 668 per cohort). Well-being was indicated by life satisfaction and depressive symptoms. Multilevel models found death-year cohort differences in life satisfaction but not depressive symptoms. While the levels of and rates of changes in life satisfaction did not differ across death-year cohorts, the association between physical health and well-being was stronger in the later-deceased cohort. These findings suggest that terminal decline in life satisfaction accelerates in interaction with disease particularly in later cohorts, with those who more recently passed away with high disease load experiencing lower levels of life satisfaction and more rapid declines. These secular trends may be due to societal advancements in healthcare systems enabling later-born cohorts to be more likely to survive with health declines.

